# Early-Onset Chronic Inflammatory Disease Associated with Maternal Microchimerism

**DOI:** 10.1155/2012/323681

**Published:** 2012-08-09

**Authors:** Tomoaki Ishikawa, Yoshihiko Sakurai, Tomohiro Takeda, Hiroshi Suzuki

**Affiliations:** ^1^Department of Pediatrics, Nara City Hospital, Nara 630-8305, Japan; ^2^Department of Pediatrics, Nara Medical University School of Medicine, Kashihara 634-8521, Japan; ^3^Department of Pediatrics, Nara Prefectural Mimuro Hospital, Sango, Nara 636-0802, Japan

## Abstract

Maternal microchimerism (mMc) refers to the presence of a small population of cells originating from the mother. Whether mMc leads to autoimmune responses in children remains controversial. We describe here an 11-year-old boy with persistent fever and elevated levels of C-reactive protein from infancy onward. During infancy, the patient presented with high fever, skin rashes, and hepatic dysfunction. Careful examination including a liver biopsy failed to reveal the cause. At 4 years old, petechiae developed associated with thrombocytopenia and positive anti-dsDNA autoantibodies. Steroid pulse therapy was effective, but the effect of low-dose prednisone was insufficient. At age 9, an extensive differential diagnosis was considered especially for infantile onset autoinflammatory disorders but failed to make a definitive diagnosis. On admission, the patient exhibited short stature, hepatosplenomegaly, generalized superficial lymphadenopathy, and rashes. Laboratory findings revealed anemia, elevated levels of inflammation markers, and hypergammaglobulinemia. Serum complement levels were normal. Serum levels of IL-6 and B-cell activating factor were elevated. Viral infections were not identified. Although HLA typing revealed no noninherited maternal antigens in lymphocytes, female cells were demonstrated in the patient's skin and lymph nodes, suggesting that maternal microchimerism might be involved in the pathogenesis of fever without source in infants.

## 1. Introduction

Maternal microchimerism (mMc) refers to the presence of a small population of cells originating from the mother. Several reports have documented that maternal microchimeric cells may be involved in the pathogenesis of some autoimmune diseases in children such as neonatal lupus [1], juvenile dermatomyositis [2], biliary atresia [3], and type 1 diabetes [4], whereas mMc may persist into adulthood without any symptoms in individuals with normal immune systems [5]. Thus, whether mMc leads to autoimmune diseases in children remains controversial [6]. Since chimerism after allogeneic HLA-mismatched stem cell transplantation can cause chronic inflammation, that is, chronic graft versus host disease (cGVHD), mMc might trigger cGVHD-like symptoms [7]. We describe a boy who had recurrent fever without source since early infancy and which was associated with mMc.

## 2. Case Presentation

An 11-year-old boy was admitted for biopsy of a skin rash and an enlarged axillary lymph node. No family history of autoimmune disease or autoinflammatory disorder was evident, and he had received no blood transfusion or stem cell transplantation. The patient had suffered from recurrent fever since infancy. At one-month old, the patient presented with fever without source, maculopapular rashes, and poor weight gain. Laboratory examinations revealed elevated levels of liver enzymes and C-reactive protein (CRP). Despite a careful examination, however, neither infection nor neoplasm was detected. There was also no evidence of biliary disorder. As the patient was well fed and his general condition was not much affected, he was followed closely as an outpatient, without therapy. Liver biopsy at seven-month old demonstrates features of chronic active hepatitis, with lymphocyte and plasma cell infiltration in Glisson's capsule yet without fibrosis and with a diffuse hepatocyte ballooning pattern.

Primary immunodeficiencies including phagocyte disorders, antibody deficiencies, complement deficiencies, and combined T- and B-cell immunodeficiencies [8] were refuted through further examinations. Liver markers gradually normalized by age 3, whereas CRP levels remained elevated, and skin rash and fever without source relapsed occasionally. This long-standing fever and elevation of CRP levels without infection or neoplasm were considered possible indicators of autoimmune disease. However, as laboratory findings supporting the diagnosis of autoimmune disease such as positive antinuclear antibody test were not obtained, a definite diagnosis could not be established. At age 4, petechiae developed on the trunk, associated with thrombocytopenia and positive anti-dsDNA autoantibodies. Intravenous pulse steroid therapy was administered and this ameliorated both the clinical and laboratory findings. Petechiae and purpura disappeared and remittent fever resolved. Platelet count increased within normal range and CRP turned negative. The elevation of anti-dsDNA autoantibodies diminished within normal limits at this time. Nevertheless, CRP level rose again soon afterwards. Low-dose oral prednisone (5 mg/day) was commenced, but high-dose prednisone was frequently required when high fever developed. Frequent exacerbations and remissions of erythematous rash were observed all the while. At age 7, investigations for short stature revealed growth hormone (GH) deficiency, and GH replacement therapy was started but showed little effect. At age 9, an extensive differential diagnosis was considered for primary immunodeficiencies, especially infantile onset autoinflammatory disorders [9, 10] with clinical manifestations even if only slightly consistent with those observed in the patient. This involved genetic testing for conditions including cryopyrin-associated periodic syndrome (C1AS1), familial Mediterranean fever (MEFV), tumor necrosis factor receptor-associated periodic syndrome (TNFRSF1A), and hyper-IgD syndrome (MVK). However, none of these genetic defects was identified. Innate immunity deficiencies were excluded as clinical symptoms of this patient were not consistent with those of defects of innate immunity currently known. The similarity of the patient's clinical course and laboratory findings to those of cGVHD suggested the stable long-term persistence of allogenic cells, that is, mMc. Throughout the course, we observed that persistent fever and elevated CRP often resolved, contrary to expectations, when the patient had symptoms of an upper respiratory tract infection. For a definitive diagnosis, a biopsy of the skin and lymph node lesions was planned.

On admission, the patient exhibited markedly short stature (−5.5 SD) unresponsive to GH treatment, hepatosplenomegaly, generalized superficial lymphadenopathy, and urticaria-like rashes. No arthropathy was observed. Laboratory findings revealed anemia (Hb 8.2 g/dL), elevated levels of inflammation markers such as CRP, ESR (79 mm/h), and SAA (563 *μ*g/dL), and hypergammaglobulinemia (IgG 3721 mg/dL). Serum complement levels were normal. Serum levels of IL-6 and B-cell activating factor (BAFF) were elevated at 21.4 pg/L and 3314 pg/mL, respectively. Viral infections such as Epstein-Barr virus, cytomegalovirus, and parvovirus B19 were not identified. Ophthalmologic examination was unremarkable. Thoracoabdominal CT scan showed hepatosplenomegaly and mild systemic lymphadenopathy. Gallium scintigraphy revealed no abnormal accumulation. Bone marrow examination revealed no abnormality. Histological examination of the skin revealed nonspecific inflammation. The perivascular and periappendicular mononuclear infiltration was observed in dermis. That of lymph node showed no increase of plasma cells but of histiocytes and dendritic cells. Epstein-Barr virus DNA and human herpes virus type 8 DNA were not detected.

**Figure 1:**
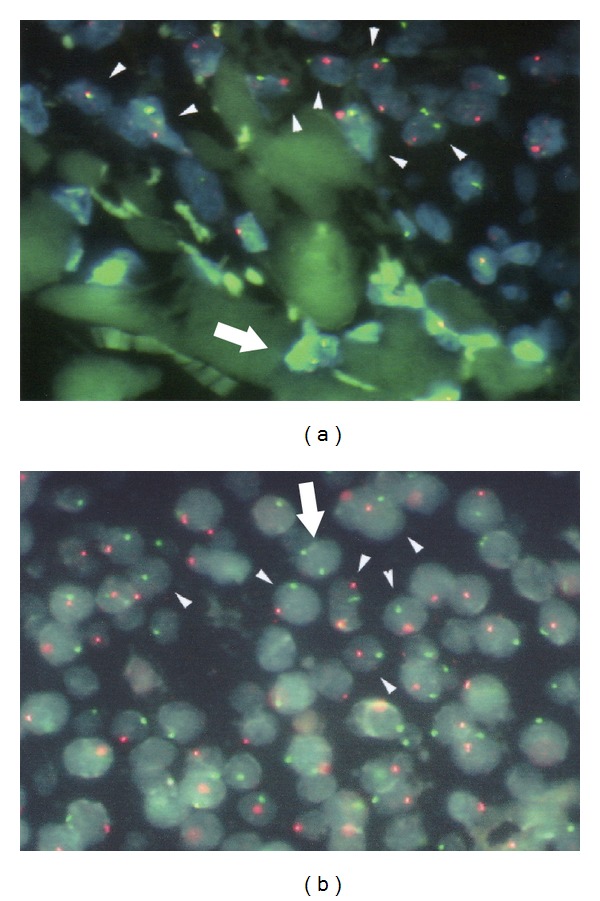
Fluorescence *in situ* hybridization analysis: maternal XX karyotype cells (arrowed lines/two green dots) among XY cells (arrowheads/red and green dots) in the skin (a) and lymph node (b).

HLA typing using nested polymerase chain reaction (PCR) with sequence-specific primer typing revealed noninherited maternal antigens below measurable limits (<0.01%) in circulating lymphocytes. On the other hand, female cells were demonstrated in the patient's skin and lymph nodes by fluorescence *in situ* hybridization labeling for the X and Y chromosomes (Figure 1). The percentage of female cells, presumed maternal, was 0.2% of all nuclei examined in the skin and 0.3% of those in the lymph node. Since elevated IL-6 was thought to be important in the pathogenesis, tocilizumab therapy was started. After the first tocilizumab infusion, IL-6 level rose further to 2230 pg/L as expected. Fever resolved, and the levels of CRP and BAFF normalized shortly thereafter. IL-6 level decreased but remained elevated (approximately 200 pg/L). The patient's height did not increase significantly, and the skin lesions persisted after more than 100 infusions.

## 3. Discussion

We present a patient with recurrent fever without source who posed a diagnostic challenge. Based on the clinical and laboratory findings, primary immunodeficiencies and autoimmune and autoinflammatory diseases had been considered in the differential diagnosis, but we had failed to make a definite diagnosis. The similarity of the affected lesions between this patient and those with cGVHD provided a clue to the involvement of mMc in the clinical condition. Although mMc exists even in normal individuals and the possibility of unknown autoinflammatory mechanisms was not entirely ruled out, several observations suggested the involvement of mMc in the etiology of this patient's underlying condition. First, quantitative analysis of microchimerism revealed female cells in the skin and lymph node lesions but not in the blood cells, suggesting the tissue-specific survival of maternal chimeric cells. In autoimmune diseases associated with microchimerism, patients with a high number of chimeric cells would be prone to enhanced immune response [11]. In the present case, the frequency of microchimeric cells was considered high, given that this has been documented at 0.05% in the skin of male infants at autopsy [12]. Secondly, BAFF level was much higher in this patient before tocilizumab infusions than those previously determined in healthy children [13]. Markedly elevated BAFF levels contribute to B-cell activation in patients with active cGVHD, which results in specific abnormalities of B-cell homeostasis [14, 15]. Thirdly, the change of the target of immune response from ever-present chimeric cells to foreign pathogen might be accountable for the resolution of fever and elevated CRP level during infectious diseases such as upper respiratory tract infections. Furthermore, the elevated levels of IL-6 in this patient were consistent with those in cGVHD [16, 17]. The rapid elevation of IL-6 levels after the first tocilizumab infusion in this patient also accords with previous observations [18]. As tocilizumab competes with IL-6 for its receptors, free IL-6 increases after infused tocilizumab occupied IL-6 receptors. Considering that free IL-6 level has not reached normal range in the present patient, however, mild inflammation might still be present. 

In conclusion, the present case suggested that tissue-specific microchimerism in which maternal cells differentiated *in situ* within fetal organs could become the target for an autoimmune response and that tocilizumab can be effective for the symptoms associated with mMc. Our experience suggests that when the origin of prolonged fever is unknown even after detailed examination, clinicians should be aware of the possible involvement of maternal microchimerism.

## References

[1] Stevens AM, Hermes HM, Rutledge JC, Buyon JP, Nelson JL (2003). Myocardial-tissue-specific phenotype of maternal microchimerism in neonatal lupus congenital heart block. *The Lancet*.

[2] Reed AM, Picornell YJ, Harwood A, Kredich DW (2000). Chimerism in children with juvenile dermatomyositis. *The Lancet*.

[3] Muraji T, Hosaka N, Irie N (2008). Maternal microchimerism in undrlying pathogenesis of biliary atresia: quantification and phenotypes of maternal cells in the liver. *Pediatrics*.

[4] Nelson JL, Gillespie KM, Lambert NC (2007). Maternal microchimerism in peripheral blood in type 1 diabetes and pancreatic islet *β* cell microchimerism. *Proceedings of the National Academy of Sciences of the United States of America*.

[5] Maloney S, Smith A, Furst DE (1999). Microchimerism of maternal origin persists into adult life. *The Journal of Clinical Investigation*.

[6] Gammill HS, Nelson JL (2010). Naturally acquired microchimerism. *International Journal of Developmental Biology*.

[7] Stevens AM (2007). Do maternal cells trigger or perpetuate autoimmune diseases in children?. *Pediatric Rheumatology*.

[8] Ten RM (1998). Primary immunodeficiencies. *Mayo Clinic Proceedings*.

[9] Geha RS, Notarangelo LD, Casanova JL (2007). Primary immunodeficiency diseases: an update from the International Union of Immunological Societies Primary Immunodeficiency Diseases Classification Committee. *The Journal of Allergy and Clinical Immunology*.

[10] Gattorno M, Federici S, Pelagatti MA (2008). Diagnosis and management of autoinflammatory diseases in childhood. *Journal of Clinical Immunology*.

[11] Evans PC, Lambert N, Maloney S, Furst DE, Moore JM, Nelson JL (1999). Long-term fetal microchimerism in peripheral blood mononuclear cell subsets in healthy women and women with scleroderma. *Blood*.

[12] Stevens AM, Hermes HM, Kiefer MM, Rutledge JC, Nelson JL (2009). Chimeric maternal cells with tissue-specific antigen expression and morphology are common in infant tissues. *Pediatric and Developmental Pathology*.

[13] Doi M, Takeda T, Sakurai Y (2010). Altered immunoglobulin A and M levels associated with changes in BAFF and APRIL after administration of intravenous immunoglobulin to treat kawasaki disease. *Journal of Investigational Allergology and Clinical Immunology*.

[14] Sarantopoulos S, Stevenson KE, Kim HT (2007). High levels of B-cell activating factor in patients with active chronic graft-versus-host disease. *Clinical Cancer Research*.

[15] Sarantopoulos S, Stevenson KE, Kim HT (2009). Altered B-cell homeostasis and excess BAFF in human chronic graft-versus-host disease. *Blood*.

[16] Imamura M, Hashino S, Kobayashi H (1994). Serum cytokine levels in bone marrow transplantation: synergistic interaction of interleukin-6, interferon-*γ*, and tumor necrosis factor-*α* in graft-versus-host disease. *Bone Marrow Transplantation*.

[17] Barak V, Levi-Schaffer F, Nisman B, Nagler A (1995). Cytokine dysregulation in chronic graft versus host disease. *Leukemia and Lymphoma*.

[18] Nishimoto N, Terao K, Mima T, Nakahara H, Takagi N, Kakehi T (2008). Mechanisms and pathologic significances in increase in serum interleukin-6 (IL-6) and soluble IL-6 receptor after administration of an anti-IL-6 receptor antibody, tocilizumab, in patients with rheumatoid arthritis and Castleman disease. *Blood*.

